# Cardiac amyloidosis and aortic stenosis: a state-of-the-art review

**DOI:** 10.1093/ehjopen/oead106

**Published:** 2023-10-12

**Authors:** Vikash Jaiswal, Vibhor Agrawal, Yashita Khulbe, Muhammad Hanif, Helen Huang, Maha Hameed, Abhigan Babu Shrestha, Francesco Perone, Charmy Parikh, Sabas Ivan Gomez, Kusum Paudel, Jerome Zacks, Kendra J Grubb, Salvatore De Rosa, Alessia Gimelli

**Affiliations:** Department of Cardiovascular Research, Larkin Community Hospital, South Miami, FL, USA; Department of Medicine, King George’s Medical University, Lucknow, India; Department of Medicine, King George’s Medical University, Lucknow, India; Department of Internal Medicine, SUNY Upstate Medical University, Syracuse, NY, USA; University of Medicine and Health Science, Royal College of Surgeons in Ireland, Dublin, Ireland; Department of Internal Medicine, Florida State University, Sarasota Memorial Hospital, Sarasota, FL, USA; Department of Internal Medicine, M Abdur Rahim Medical College, Dinajpur, Bangladesh; Cardiac Rehabilitation Unit, Rehabilitation Clinic ‘Villa delle Magnolie’,81020 Castel Morrone, Caserta, Italy; Carle BroMenn Medical Center, Normal, IL, USA; Department of Cardiovascular Research, Larkin Community Hospital, South Miami, FL, USA; Department of Medicine, Kathmandu University School of Medical Science, Dhulikhel, Kathmandu 45209, Nepal; Department of Cardiology, The Icahn Medical School at Mount Sinai, NewYork 10128, USA; Division of Cardiothoracic Surgery, Department of Surgery, Emory University School of Medicine, Atlanta, GA, USA; Department of Medical and Surgical Sciences, Magna Graecia University, Catanzaro, Italy; Department of Imaging, Fondazione Toscana/CNR Gabriele Monasterio, Pisa 56124, Italy

**Keywords:** Cardiac amyloidosis, Aortic stenosis, Cardiomyopathy, Diagnosis

## Abstract

Cardiac amyloidosis is caused by the extracellular deposition of amyloid fibrils in the heart, involving not only the myocardium but also any cardiovascular structure. Indeed, this progressive infiltrative disease also involves the cardiac valves and, specifically, shows a high prevalence with aortic stenosis. Misfolded protein infiltration in the aortic valve leads to tissue damage resulting in the onset or worsening of valve stenosis. Transthyretin cardiac amyloidosis and aortic stenosis coexist in patients > 65 years in about 4–16% of cases, especially in those undergoing transcatheter aortic valve replacement. Diagnostic workup for cardiac amyloidosis in patients with aortic stenosis is based on a multi-parametric approach considering clinical assessment, electrocardiogram, haematologic tests, basic and advanced echocardiography, cardiac magnetic resonance, and technetium labelled cardiac scintigraphy like technetium-99 m (^99m^Tc)-pyrophosphate, ^99m^Tc-3,3-diphosphono-1,2-propanodicarboxylic acid, and ^99m^Tc-hydroxymethylene diphosphonate. However, a biopsy is the traditional gold standard for diagnosis. The prognosis of patients with coexisting cardiac amyloidosis and aortic stenosis is still under evaluation. The combination of these two pathologies worsens the prognosis. Regarding treatment, mortality is reduced in patients with cardiac amyloidosis and severe aortic stenosis after undergoing transcatheter aortic valve replacement. Further studies are needed to confirm these findings and to understand whether the diagnosis of cardiac amyloidosis could affect therapeutic strategies. The aim of this review is to critically expose the current state-of-art regarding the association of cardiac amyloidosis with aortic stenosis, from pathophysiology to treatment.

## Graphical Abstract

**Graphical Abstract:**
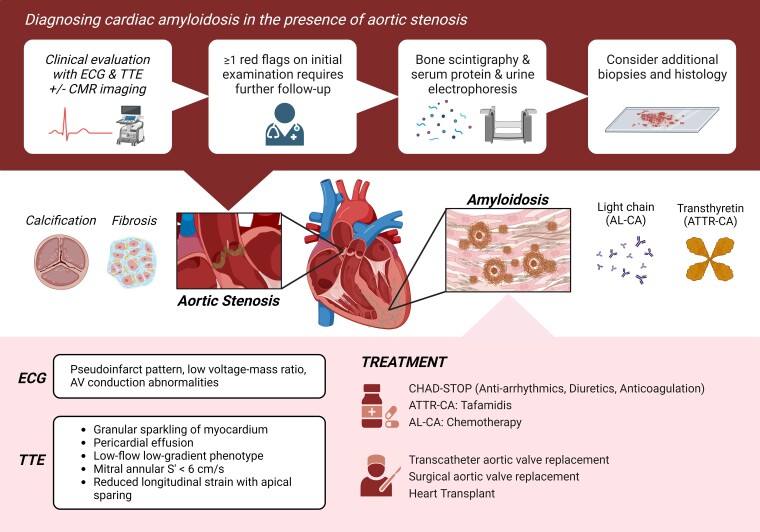
Cardiac amyloidosis and aortic stenosis: diagnostic imaging, clinical presentation, and management criteria. ATTR-CA, transthyretin cardiac amyloidosis; AL-CA, amyloid light chain–cardiac amyloidosis; AV, aortic valve; CMR, cardiac magnetic resonance; ECG, electrocardiogram; TTE, transthoracic echocardiogram.

**Highlights**

- Coexistence of cardiac amyloidosis and aortic stenosis is common, but the diagnosis of cardiac amyloidosis in the presence of aortic stenosis presents a challenge due to the overlapping clinical features. This literature review emphasizes the clinical characteristics that may suggest the presence of concomitant cardiac amyloidosis.
- Recent advancements in echocardiography and cardiovascular magnetic resonance imaging have provided promising findings. These imaging modalities have shown significant differences in diastolic, right ventricular, and left ventricular function between patients with concomitant cardiac amyloidosis and aortic stenosis.
- Radionuclide imaging is also an effective tool for distinguishing between the two conditions and providing valuable insights for guiding appropriate treatment. With its ability to assess myocardial function, identify the presence of cardiac amyloidosis, and evaluate the severity of aortic stenosis, radionuclide imaging offers a comprehensive evaluation of these complex conditions. By leveraging this valuable information, physicians can make informed decisions and tailor treatment plans to the individual needs of each patient.

## Introduction

Cardiac amyloidosis (CA) is caused by the extracellular deposition of misfolded proteins, known as amyloid, in the heart. Over 30 proteins are known to cause amyloidosis; however, transthyretin CA, i.e. ATTR-CA, has been explored in much greater detail.^1,2^ The ATTR-CA was previously classified as a rare form of infiltrative cardiomyopathy; however, recent advances in diagnostic imaging have led to a significant increase in disease awareness and diagnosis in the past decade. Infiltration of this protein can cause the ventricular walls to thicken and interfere with the heart's conduction system, leading to arrhythmias and reduced cardiac output. Cardiac involvement is the primary cause of death in systemic amyloidosis cases.^3^

Aortic stenosis (AS) is a common condition that primarily affects older adults and can be caused by age-related degeneration, rheumatic fever, or congenital malformations. The disease leads to pressure and volume changes in the left ventricle (LV) due to increased afterload,^4^ and symptoms typically occur when the valvular obstruction becomes severe. Whilst aortic valve replacement (AVR) can be effective in treating AS, the condition can still lead to a high risk of morbidity and a 50% risk of mortality.^5^ Recent studies have found that CA and AS can coexist in older patients (>65 years), with ATTR-CA occurring in approximately 4–16% of AS cases, especially those who undergo transcatheter AVR (TAVR).^2^

The prevalence of both ATTR-CA and AS disease entities increases with age, with several studies reporting ATTR-CA in patients with severe AS. As such, the clinical implications of this association raise the question of whether AS severity plays a role in detecting underlying ATTR-CA.^2^ The haemodynamic changes seen in AS, including reduced cardiac output due to ventricular remodelling and diastolic dysfunction, resemble the restrictive cardiomyopathy seen in CA, which can complicate diagnosis and management.^6^ Therefore, it's important to standardize and improve the diagnostic assessment and therapeutic management of CA in AS. This review aims to provide a comprehensive overview of CA coexisting with AS, with an emphasis on the diagnostic modalities available. We also propose algorithms based on available studies that shall guide clinicians to suspect, diagnose, and treat concomitant CA accurately.

## Pathophysiology of cardiac amyloidosis

Out of the 30 proteins linked to amyloid aggregation, only nine are known to accumulate in varying degrees in the myocardium, the most common being the amyloid light chain (AL) (heart involvement in 70% of cases), ATTR variant (ATTRv) (heart involvement in 30–100% of cases depending on the mutation), or wild-type ATTR (ATTRwt) (100% of cases involve heart).^7^ The median survival rate after diagnosis is lowest for the AL subtype, at 24 months [or 6 months if heart failure (HF) at diagnosis is not treated].^7^ The average age of CA patients ranges from 74 to 90 years, with most diagnoses occurring in women in their eighth or ninth decade.^8^

**Figure 1:**
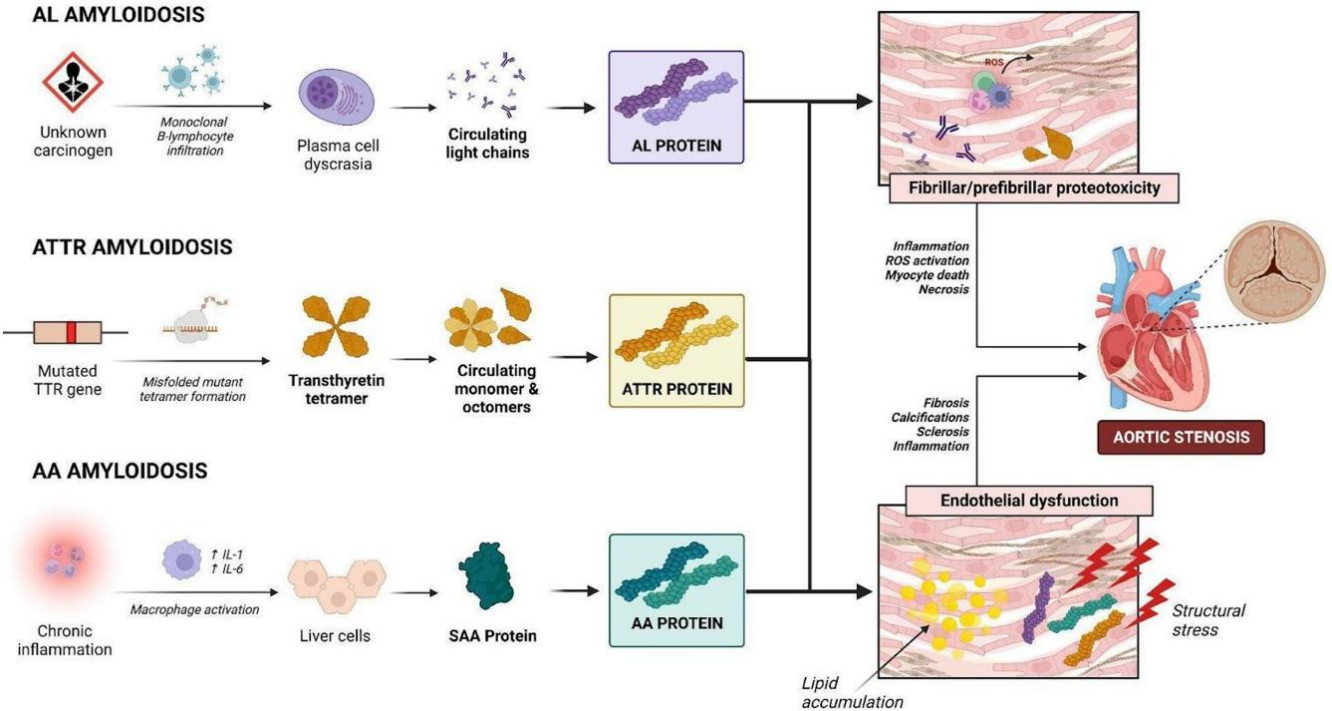
Pathophysiology of amyloidosis and its effects on the development and progression of aortic stenosis. AA amyloidosis, secondary amyloidosis; AL amyloidosis, amyloid light chain amyloidosis; ATTR amyloidosis, transthyretin amyloidosis; IL-1, interleukin-1; IL-6, interleukin-6; ROS, reactive oxygen species; SAA, serum amyloid A protein; TTR, ttransthyretin.

Two of the most persistent sites of amyloid accumulation are the cardiac valves and myocardium.^9^ In the AL subtype, the accumulation of monoclonal B cells resulting from exposure to different carcinogens promotes plasma cell dyscrasia, which raises the level of immunoglobulin light chains in the plasma. The ATTR subtype is believed to result from a gene misfolding mutation that causes the formation of a transthyretin tetramer. Inflammation due to lipid infiltration and structural stress results in endothelial deformity (as shown in *Figure 1*), which causes calcification, stiffness, fibrosis, and sclerosis of the aortic valve leaflets, leading to a progressive decline in pressure beyond the valve.^10,11^ Amyloid infiltration can worsen AS by causing pressure overload, as evidenced by a high prevalence of amyloid infiltrates in surgically removed heart valves.^9^

## Diagnosis of amyloidosis in the setting of aortic stenosis

The diagnosis of amyloidosis presents multi-faceted challenges, often resulting in its underestimation in current clinical practice worldwide. This oversight can be attributed to several factors. Firstly, the comprehensive description of cardiac manifestations in amyloidosis remains limited and is a continually evolving field. Moreover, a definitive diagnosis requires advanced imaging techniques like nuclear imaging or cardiac MRI, as well as invasive procedures such as endomyocardial biopsy. In contrast, AS is a well-recognized valvular disorder that predominantly affects the elderly, with diagnosis and staging primarily reliant on echocardiographic evaluation. Due to its higher prevalence and well-established diagnostic protocols, clinicians are adept at identifying AS, even in its milder forms. However, challenges arise when both conditions coexist within a patient. The presence of AS can obscure the distinct findings of CA, potentially leading to a significant number of cases going undetected, given the limited utilization of advanced diagnostic tools for AS and their availability in select centres.

**Figure 2:**
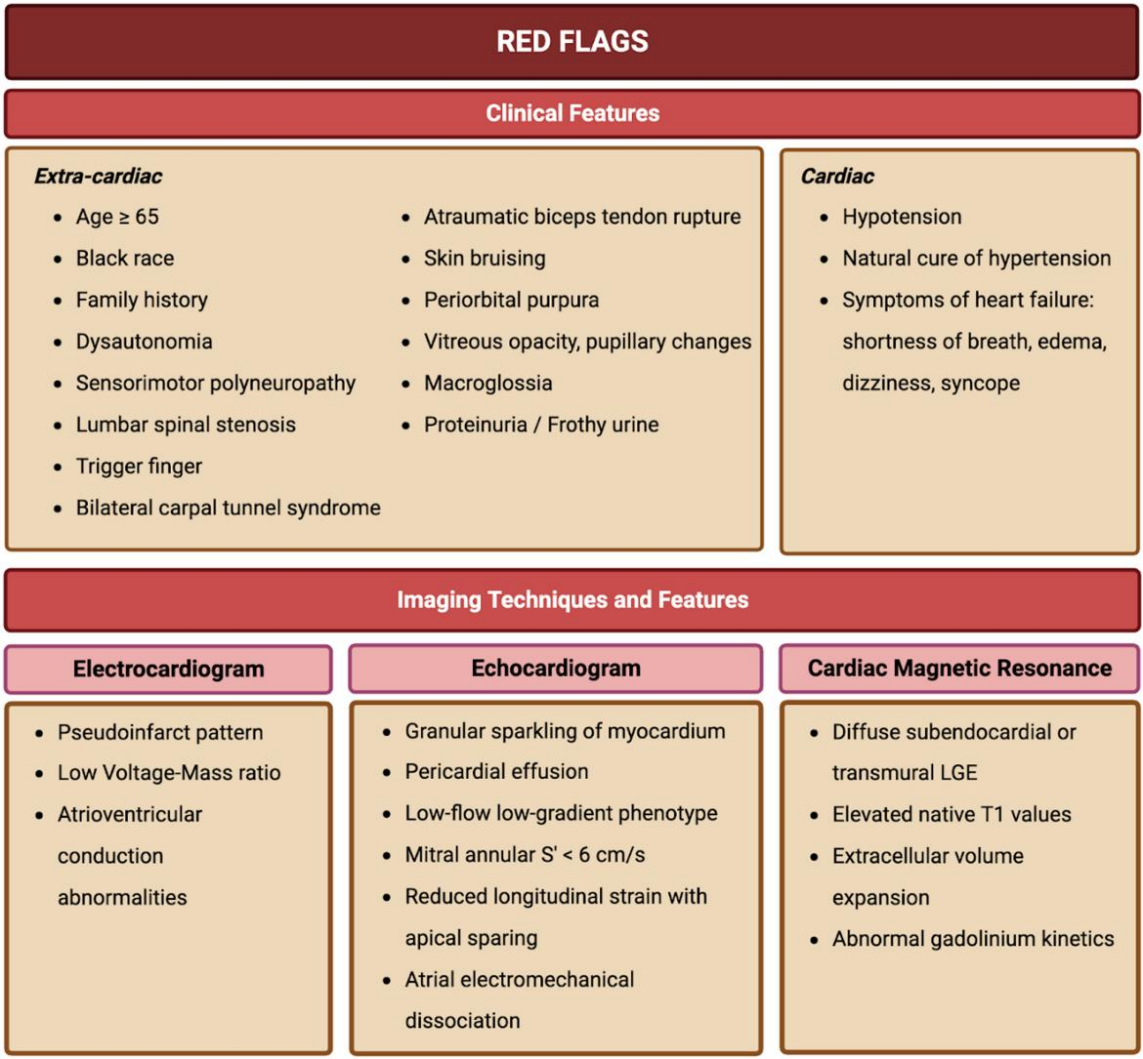
Clinical ‘red flags’ suspecting of cardiac amyloidosis in aortic stenosis. LGE, late gadolinium enhancement.

**Figure 3:**
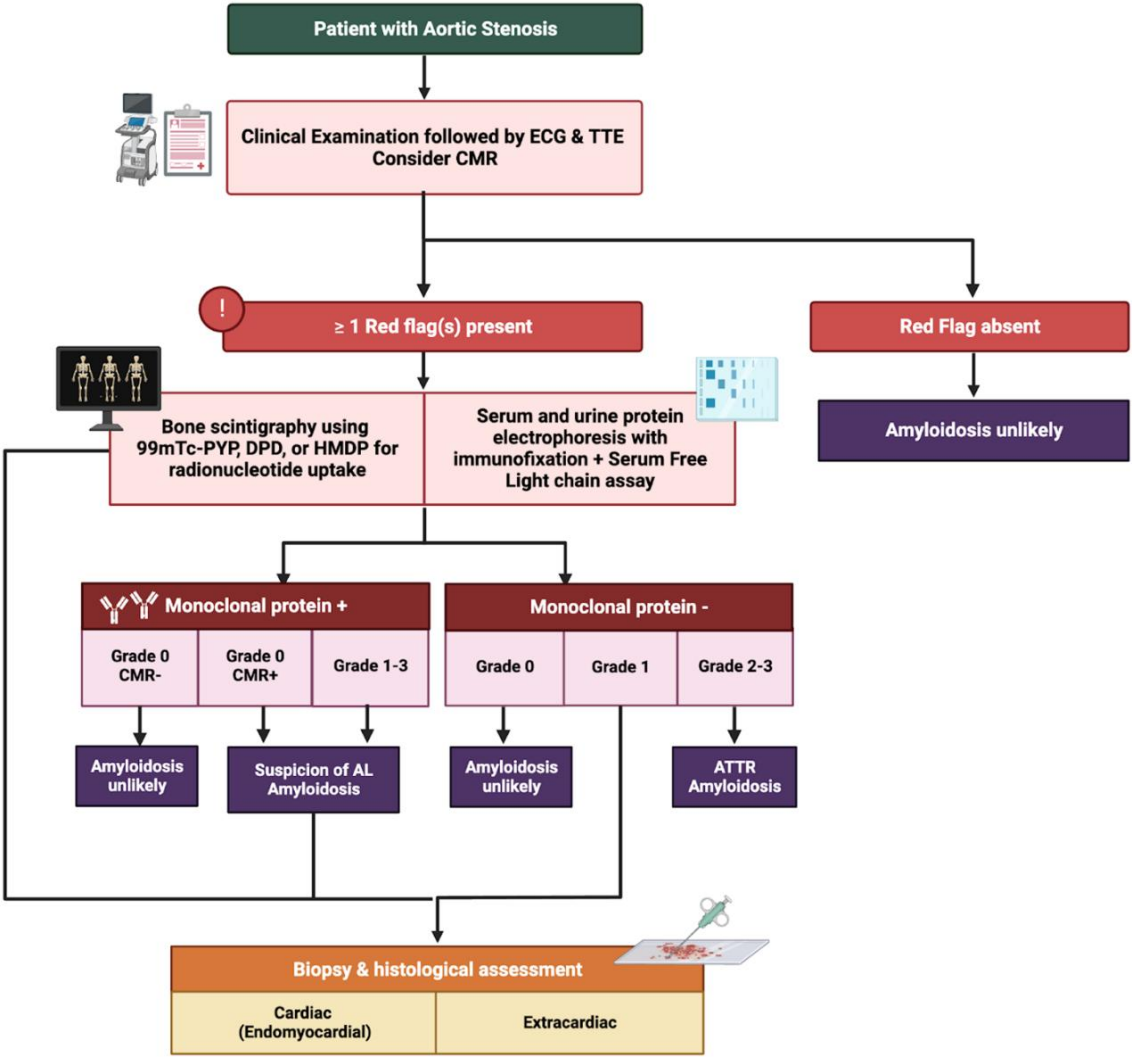
Diagnostic algorithm of amyloidosis in patients with confirmed aortic stenosis. ATTR, transthyretin amyloidosis; CMR, cardiac magnetic resonance imaging; DPD, 3,3-diphosphono-1,2-propanodicarboxilic acid; ECG, electrocardiography; HMDP, hydroxymethylene diphosphonate; PYP, pyrophosphonate; TTE, transthoracic echocardiography.

Detecting CA requires a heightened sense of suspicion, as routine tests fall short of yielding a definitive diagnosis. Whilst numerous reviews have concentrated on formulating diagnostic algorithms for lone CA, predominantly centred around advanced imaging techniques, it is crucial to underscore the significance of observations gleaned from routine assessments. This is particularly pertinent as advanced testing might remain unexplored without a sufficiently elevated index of suspicion. This review endeavours to synthesize existing literature, elucidating the significance of coexisting AS and CA in routine investigations like electrocardiogram (ECG) and transthoracic echocardiography (TTE). By doing so, it aims to empower clinicians with the ability to foster early clinical suspicion.^12^ This initial phase becomes pivotal in paving the way for the deliberate implementation of advanced diagnostic modalities like cardiac magnetic resonance and cardiac scintigraphy that might otherwise be omitted (the diagnostic algorithm has been illustrated in *Figures 2* and *3*). Ultimately, this comprehensive approach augments the prospects of achieving a timely and precise diagnosis.

The coexistence of AS and CA has been found to be more common in older age groups and males.^13^ Clinical features suggestive of CA include carpal tunnel syndrome, stenosis of the lumbar spine, biceps tendon rupture, deafness, and symptoms of HF. Macroglossia is also commonly associated with AL-CA.^7,14,15^ Autonomic and peripheral neuropathy is associated with CA (mainly ATTR-CA), which also leads to a decrease in blood pressure.^15^ Progressive deposition of amyloid also disrupts the conduction system, leading to dysfunction of the sinoatrial node, atrial flutter, and bundle branch block.^7,13,15^ Features of AS found in patients of CA include low-flow, low-gradient haemodynamics, amyloid infiltration in the aortic valve, and faster progression of AS.^14,16^ In patients with coexistent AS-CA, poorer performance was found on the 6-min walk test than in patients with AS alone (Clinical ‘red flags’ of coexistent AS-CA have been enlisted in *Figure 2*. Evidence of myocardial injury has been found in up to 80% of CA patients. However, the exact incidence may vary based on the type of amyloidosis and the population studied, the disease stage, and ethnicity^17^ similar to what was observed with acute coronary syndromes or HF.^18^

### Biomarkers

Natriuretic peptides, especially brain natriuretic peptides (BNPs), have been found very useful in diagnosing ventricular dysfunction. In cases of CA, BNP levels are elevated in ventricular myocytes in areas adjacent to amyloid deposition.^19^ In patients with biopsy-confirmed systemic amyloidosis, the levels of N-terminal fragment of the pro-BNP (NT-proBNP) > 332 pg/mL in the absence of renal failure or atrial fibrillation (AF) or both are indicative of cardiac involvement.^20^ It has also been found that amongst the two major types of CA, circulating levels of NT-proBNP are higher in cases of AL-CA, which may be attributed to the toxic effect of light chain.^21^ Cardiac troponins T and I (cTnT and cTnI) have been well established as markers of cardiac injury. Though there has not been much reporting on the diagnostic role of cardiac troponin in CA, it has been shown that levels of high sensitive cTnT (hs-cTnT) are significantly higher in patients of CA [0.048 (0.029–0.073) vs. 0.016 (0.010–0.031) ng/mL; *P* < 0.001]; the same study reported log hs-cTnT to be an independent diagnostic tool in CA.^22^ Both NT-proBNP and cardiac troponins are used to formulate staging scores for AL-CA and ATTR-CA.^23^ However, it should be considered that an elevation of serum troponin can be the first clinical presentation of CA, but it has low specificity as it can be associated with a number of alternative diagnoses, such as coronary syndromes, AS, or HF.^24^ Release kinetics are usually useful to differentiate the diagnoses underlying myocardial injury. In addition to the simple evaluation of cardiac necrosis marker trends, mathematical models are currently available to improve the discrimination capacity.^25–27^

The traditional approach to diagnosing AL amyloidosis is serum and urine protein electrophoresis (SPEP and UPEP), wherein the appearance of an M spike is suggestive of monoclonal gammopathy. However, it's important to note that a wide range of haematological conditions like multiple myeloma, chronic myeloid leukaemia, chronic lymphocytic leukaemia, Waldenström’s macroglobulinaemia, etc. can also cause an M spike on electrophoresis. For this reason, serum-free light chain (sFLC) assay is also used as a preliminary diagnostic tool for CA. It measures the relative levels of circulating kappa and lambda light chains, with a normal range of 0.26–1.65. However, sFLC ratios can be non-specific, so they must be interpreted carefully. About 20–40% of patients with ATTR-CA also have an unrelated monoclonal gammopathy of undetermined significance,^28^ so sFLC cannot be the sole diagnostic for CA. Protein electrophoresis alone is also not enough for diagnosing AL-CA, as approximately 30% of patients with normal results have abnormal immunofixation electrophoresis (IFE).^29^ Therefore, PEP should be accompanied by serum and urine IFE and sFLC for a combined sensitivity of 99%.^28,30^ These biomarkers can also be used to categorize patients by risk and monitor their response to treatment.

### Electrocardiogram

Patients diagnosed with CA typically do not exhibit a standard ECG. Low voltage complexes on ECG, characterized by a QRS amplitude of less than 1 mV in all precordial leads or less than 0.5 mV in all limb leads, have traditionally been considered a pathognomonic feature of CA. Nevertheless, the reported prevalence of low voltage complexes varies considerably across studies, ranging from 20% in ATTR-CA to 60% in AL-CA.^31,32^ The Sokolow–Lyon index (S wave in lead V1 + R wave in lead V5 or V6 < 1.5 mV) has been associated with negative outcomes in CA^31^ but may not be useful for early detection since low voltage complexes appear later in the disease process.^31–34^ Amyloid light chain–cardiac amyloidosis tends to exhibit lower voltage complexes compared with ATTR-CA, possibly due to their distinct disease progression patterns.^32,33^

Infiltrative cardiomyopathies such as CA are distinguished by a low voltage-to-mass ratio, which has been demonstrated to be more sensitive than low-voltage alone. The voltage-to-mass ratio is calculated as the Sokolow–Lyon index divided by the cross-sectional area of the LV wall, and a ratio of less than 1.5 is indicative of CA.^35^ Patients with concurrent CA and AS were observed to have a lower voltage-to-mass ratio in comparison with those with just AS.^36,37^ Moreover, patients with ATTR-CA had a higher mean voltage-to-mass ratio compared with those with AL-CA (1.1 ± 0.5 in ATTR-CA vs. 0.9 ± 0.5 in AL-CA; *P* < 0.0001).^32^

In addition to voltage alterations, the most prevalent ECG finding in CA patients is a pseudo-infarction pattern, which primarily appears in the anterior leads.^31^ A pseudo-infarct pattern is defined by pathological Q waves (1/4 of R amplitude) or QS waves in two consecutive leads in the absence of LV wall dynamics anomalies, ischaemic heart disease, and left bundle branch block.^34^ This pattern is often seen in both ATTR-CA and AL-CA cases and has been shown to be linked with worse outcomes in AL-CA patients.^37–39^ Amyloid accumulation in the microcirculation and smaller intramyocardial arteries are likely the cause of aberrant Q waves on the ECG in those who lack an overt epicardial coronary occlusion.

Patients with both concomitant AS and CA may experience conduction anomalies, which can affect the sinoatrial node, the atrioventricular node, and the bundle branches to varying degrees. Such anomalies are typically more significant in patients with both conditions than in those with only AS. In a study by Castaño *et al*.,^6^ individuals with concurrent ATTR-CA and AS showed longer QRS duration (127 ms vs. 110 ms; *P* = 0.017) and a higher prevalence of right bundle branch block (37.5% vs. 15.8%; *P* = 0.023) compared with those with only AS. Conduction abnormalities can often result in arrhythmia, which is a common co-morbidity in patients with AS, such as AF. Interestingly, AF is also prevalent in approximately 50% of all CA patients, and its occurrence is more frequent in ATTR-CA than in AL-CA.^40–42^ In another study that reported on individuals with both AS and ATTR-CA, 42–67% of patients had AF.^2,6,34,43^ However, the prevalence of atrial arrhythmias was significantly higher in patients with dual pathology: 67% in the concurrent ATTR-CA group vs. 20.2% in the isolated AS group (*P* = 0.006).^43^

As the degree of myocardial amyloid deposition can range from minimal to transmural, the prevalence of ECG abnormalities discussed above tends to increase with the severity of the disease.^2,44^ Therefore, whilst various ECG abnormalities may suggest the presence of CA, they are not specific on their own. In conjunction with other compatible clinical indications, the presence of a pseudo-infarct pattern without a history of myocardial infarction, low ECG voltage despite LV hypertrophy (LVH), conduction abnormalities (especially right bundle branch block), and atrial arrhythmias should raise suspicion for the possibility of CA.

### Echocardiogram

Transthoracic echocardiography is the first test in a series of tests for the diagnosis of amyloidotic cardiomyopathy. Transthoracic echocardiography provides clinicians with a non-invasive diagnostic and prognostic modality for AS-CA as it is safe to use, readily available, portable, and feasible and provides haemodynamic and diastolic function assessments. Echocardiographic scores based on valvular characteristics have demonstrated reasonable diagnostic performance to differentiate ATTR- from AL-CA and ATTR-CA from their matched controls.^45^ However, CA is an infiltrative disorder that impacts all the chambers of the heart. Therefore, a thorough echocardiographic assessment is likely to enhance clinical suspicion and diagnostic accuracy.

#### Left ventricular remodelling and systolic dysfunction

Both AS and CA have traditionally been associated with concentric hypertrophy of the LV.^46^ On the contrary, studies have shown that as many as one-third of AL-CA cases have a normal LV wall thickness of 12 mm or less^47^ and as many as 79% of ATTR-CA patients have asymmetrical septal hypertrophy.^48^ Despite this, a recent meta-analysis demonstrated that echocardiographic parameters indicative of concentric hypertrophy, such as increased LV mass index, interventricular septal thickness, posterior wall thickness, and relative wall thickness (RWT), are reliable markers for identifying CA, even in the presence of AS.^49^ However, it is worth noting that amongst these indices, only the LV mass index has been identified as a predictor of mortality.^43,50–52^

Patients with both AS and CA have lower indicators of systolic dysfunction, including LV ejection fraction (LVEF), stroke volume index (SVi), myocardial contraction fraction (MCF), and mitral annular S’ compared with those with AS alone^49,53^ Patients with AS-CA have a significantly reduced LVEF compared with those with AS alone [pooled standardized mean difference (SMD): −0.46 (−0.81 to −0.11)].^49^ Considering that in the absence of coronary artery disease (CAD), the LVEF remains preserved in AS, and the concomitant presence of severe AS and reduced LVEF is relatively uncommon^54^ such a finding should raise suspicion about the coexistence of CA. Furthermore, the SVi has also been identified as a predictor of mortality^43,51^ and is significantly reduced in AS-CA.^6,36,43,51^ However, it is important to consider that these indices might be affected by the marked LVH that occurs in AS-CA. There is a distinct correlation between LVEF and RWT. As a result, the LVEF tends to rise proportionally with the extent of LV concentric remodelling. Therefore, the LVEF can remain preserved in AS-CA despite reduced myocardial contractility, making it challenging to interpret LVEF or SVi as reliable markers of LV dysfunction.

Myocardial contraction fraction has emerged as a new volumetric measure of LV contractility that highlights anomalies in myocardial shortening that may be concealed by preserved EF. It is easily obtainable through routine Doppler echocardiography by the ratio of LV stroke volume to myocardial volume. In patients with low gradient (<40 mm Hg) severe AS (aortic valve area ≤ 1 cm^2^) with preserved EF (≥50%), MCF has been demonstrated as a valuable indicator for predicting mortality risk, particularly in patients with normal flow (SVi ≥ 30–35 mL/m^2^).^55,56^ Interestingly, it is also significantly reduced in CA and more strongly related to mortality than EF in both AL-CA^57^ and ATTR-CA.^52^ Therefore, it would not be surprising to observe an additive effect in cases of AS-CA. Indeed, a meta-analytical comparison of MCF in patients with isolated AS vs. AS-CA revealed that MCF was significantly lower in the latter [SMD: −2.88 (−5.70 to −0.06)].^49^ Similarly, the average mitral annular S’ is also significantly reduced in AS-CA,^49^ and a cut-off of less than 6 cm/s is particularly sensitive for diagnosing ATTR-CA in patients with severe AS.^16^

Patients who have both concomitant CA and AS are more likely to present with a low-flow, low-gradient phenotype in terms of AS severity. More than 75% of the patients in a study by Cavalcante *et al*.^43^ who had both ATTR-CA and AS showed this pattern. Three further studies found comparable results.^6,58,59^ However, it is challenging for clinicians to determine the severity of AS in patients with low-flow low-gradient, and additional imaging tests are required to differentiate between true-severe and pseudo-severe AS.^16^ Assessment of myocardial deformation parameters (such as strain, strain rate, or twist) can provide better quantitation of regional and global systolic function and has proved beneficial in assessing AS severity.^37^ There has been a growing interest in the use of strain imaging to diagnose patients with suspected CA, as studies have shown that a reduction in peak systolic global longitudinal strain (GLS) can be detected even before the conventional echocardiographic parameters become abnormal.^60–63^ Castaño *et al*.^6^ demonstrated a significantly reduced GLS in patients with AS-CA as compared with lone AS. However, GLS reduction is not unique to AS-CA, as any disease that reduces EF can cause a decrease in GLS. To overcome this limitation, regional strain assessment using speckle-tracking echocardiography has demonstrated high sensitivity and specificity (93% and 82%, respectively) for diagnosing CA and differentiating it from other causes of LVH.^61^ One of the most significant predictors of CA is impaired mid and basal LS of the LV, with the apex being relatively spared, possibly due to lower extracellular amyloid deposition, which results in higher myocardial contraction and less resistance to deformation at the apex compared with other segments. This finding is also common in patients with AS^64^ regardless of CA, likely due to haemodynamic stress and increased afterload from the stenotic aortic valve. Jaiswal *et al*.^49^ found that the relative apical LS was not significantly different between the two groups of patients and validated this conclusion.

The dependence of GLS on afterload represents another limitation in severe AS. In this regard, myocardial work (MW) is a non-invasive measure of myocardial performance based on LV strain assessment (estimated as the area of the LV pressure–strain loop), which may be useful in predicting non-responders or differentiating those with a low-flow, low-gradient pattern in patients with severe AS.^65^ Additionally, non-invasive MW indices are significantly altered in patients with CA^66^ and have prognostic and predictive potential in this context.^67^ Therefore, non-invasive MW measurement may be valuable in the management of patients with both AS and CA.

#### Diastolic dysfunction and left atrial remodelling

Both CA and AS are associated with diastolic dysfunction characterized by abnormalities in the relaxation and filling of the ventricles. However, the severity of diastolic dysfunction may be greater in patients with both AS and CA, as evidenced by the statistically significant differences in early-filling-velocity to atrial-filling-velocity ratio (*E*/*A* ratio) and left atrial (LA) size.^49^ Whilst LVH and fibrosis are the primary cause of diastolic dysfunction in AS, this is further exaggerated by the progressive infiltration of amyloid fibrils in AS-CA, ultimately leading to a restrictive filling pattern.

The increased *E*/*A* ratio in CA can be attributed to various factors. Hypotheses proposed by Plehn *et al*.^68^ suggest that this phenomenon may arise from atrial systolic failure, an outcome of amyloid infiltration within the atrial tissue. However, investigations highlighting prominent atrial reversal flow velocities in the pulmonary vein challenge this notion, pointing instead towards diminished LV compliance during atrial contraction, signifying augmented atrial afterload as the potential underlying cause rather than intrinsic atrial failure.^69^ A progressive continuum of impaired diastolic filling abnormalities in both the right ventricle (RV) and LV has been postulated. In the early stages of the disease, a prolonged or impaired relaxation of the LV shifts the diastolic filling to late diastole, accompanied by heightened LA contribution as a compensatory response. This initial phase exhibits Doppler flow patterns akin to other conditions, such as hypertension, CAD, hypertrophic cardiomyopathy (HCM), and aging.^69^ As the disease advances, there is a greater infiltration of amyloid fibrils in the myocardium, which confers a highly rigid ventricular structure, ultimately restricting filling and causing a significant increase in ventricular pressure for only a minimal change in volume. Some patients may exhibit a pseudo-normal phase, denoting a transition from abnormal relaxation towards restriction as LA pressure increases. It can be challenging to discern whether a diminutive transmitral A wave is indicative of true atrial contractile dysfunction or reflective of restrictive LV pathophysiology or both.

Patients with CA are at an elevated risk of developing AF and thromboembolic events. Recent research focusing on amyloidosis patients has underscored the connection between LA dysfunction and all-cause mortality.^70,71^ Given the pronounced diastolic dysfunction commonly seen in CA, the loss of the atrial contribution to ventricular filling has profound haemodynamic implications, leading to clinical deterioration and hospitalizations. Therefore, it becomes imperative to assess LA dysfunction in these patients. Left atrial volume is widely recognized as a valuable indicator reflecting the cumulative impact of prolonged elevation in LV filling pressure, serving as a reflection of the seriousness and duration of diastolic dysfunction. In particular, the LA volume index (LAVi) holds greater prognostic significance than being a marker of atrial dysfunction. In AL amyloidosis cases, it stands out as an independent predictor of mortality. Geenty *et al*.^72^ introduced an echocardiographic scoring system that combines both LA volume and LVGLS, demonstrating a comparable ability to predict all-cause mortality as the Mayo stage.

Echocardiographic speckle-tracking has now firmly established its role in the evaluation of atrial function. It enables the assessment of various facets of atrial function, including the ability of the atria to expand during ventricular systole (known as reservoir function), early diastolic emptying (conduit function), atrial shortening (atrial contraction), and, when coupled with LV pressure estimates, the LA's resistance to deformation (referred to as LA stiffness). Initial findings have indicated compromised atrial systolic contraction and a decline in reservoir function, resulting in an atrium primarily functioning as a conduit throughout the cardiac cycle—commonly termed restrictive atrial dysfunction.^70,73–75^ Left atrial strain parameters exhibit a strong correlation with LVGLS and can serve as an alternate marker for elevated filling pressure, illustrating the LA's compensatory role in maintaining normal LV filling pressure.^74,76^ Furthermore, these parameters hold diagnostic significance, aiding in the differentiation between CA and other causes of HCM.^77–79^ Notably, ATTR-CA patients tend to exhibit lower LA reservoir function when compared with their counterparts with AL-CA.^75^

The aberrant stiffness of the LA myocardium can also manifest in atrial electromechanical dissociation.^70,80,81^ Intriguingly, a subset of patients in sinus rhythm exhibits no discernible evidence of atrial mechanical contraction on strain analysis, notwithstanding the presence of P waves on the ECG.^70^ Atrial electromechanical dissociation is associated with unfavourable clinical outcomes, akin to patients with AF, and may elevate the risk of thromboembolism, further underscoring its clinical significance.^80,81^

#### Right ventricular dysfunction

Patients with either AS or CA also exhibit varying degrees of RV impairment. Both tricuspid annular plane systolic excursion and tricuspid annular S’, which represents RV systolic function, were significantly reduced in CA-AS patients [pooled SMD = −0.36 (−0.62 to −0.09) and −0.77 (−1.13 to −0.42), respectively] implying a more significant RV dysfunction in this group of patients.^49^ Tricuspid annular plane systolic excursion with a cut-off of ≤19 mm has been proposed as part of a scoring system alongside other indicators to detect AL-CA and ATTR–CA with high accuracy in individuals with suspected CA (area under curve [AUC]: 0.90 and 0.87, respectively).^82^

### Cardiac magnetic resonance

Cardiovascular magnetic resonance (CMR) is currently considered the most effective non-invasive imaging technique for assessing cardiac function and myocardial tissue characterization. Cardiovascular magnetic resonance offers high spatial and temporal resolution and inherent blood-to-tissue contrast, making it an ideal diagnostic method for identifying cardiomyopathies. The use of late gadolinium enhancement (LGE) imaging allows for the visualization of cardiac fibrosis. The latest generation of CMR development, known as CMR parametric mapping, includes T1-, T2-, and extracellular volume (ECV)-mapping, which provides a pixel-by-pixel representation of absolutely quantized numerical T1 or T2 features expressed in units of time (e.g. milliseconds).^83^ This is in contrast to traditional T1- or T2-weighted MRI techniques that rely on relative imaging signal intensities to highlight and differentiate abnormal from normal tissue areas.

Cardiovascular magnetic resonance also plays a crucial role in assessing severe AS patients eligible for transcatheter aortic valve implantation (TAVI). Cardiovascular magnetic resonance provides accurate anatomical evaluations and functional assessments of the LV, aiding in interventional planning. Whilst computed tomography (CT) excels in assessing calcification and vessel size, CMR demonstrates higher sensitivity and specificity in identifying valve pathology. Additionally, CMR's ability to detect myocardial fibrosis contributes to prognostic value in AS patients, optimizing the timing of AVR for improved outcomes.^84^

T1 relaxation time, also known as spin-lattice relaxation time, is a decay constant that controls the return of longitudinal magnetization to its thermal equilibrium after being disturbed by an electromagnetic pulse.^83^ The Society for Cardiovascular Magnetic Resonance and the CMR Working Group of the European Society of Cardiology recommend incorporating T1 mapping into standard CMR practice as a key tissue characterization technique.^85^ Non-contrast T1 mapping correlates well with systolic and diastolic dysfunction markers and offers a good diagnostic sensitivity for identifying CA.^85^ It can effectively identify and quantify cardiac involvement at the level of myocardial infiltration seen in amyloidosis, making it a potentially valuable diagnostic tool. Furthermore, the specificity of the approach was demonstrated by showing that the T1 elevation in amyloidosis with considerable cardiac involvement was higher than in other myocardial diseases with equivalent hypertrophy.^85^ In a study by Cavalcante *et al*.,^43^ patients with dual pathology exhibited significantly higher values of T1 than the patients with AS alone (1125 ± 49 vs. 1035 ± 60; *P* = 0.002). These higher T1 values could be due to a higher proportion of amyloid infiltration for a given wall thickness compared with fibrosis or a more significant amyloidosis-induced T1 prolongation compared with myocardial fibrosis.^86^

Cardiovascular magnetic resonance with LGE offers unique information on myocardial tissue characterization. The theory behind post-contrast T1 is that gadolinium administration leads to a shortening of the T1 time. A decreased post-contrast T1 value indicates an increase in interstitial space as gadolinium agents are mostly distributed in the intravascular and interstitial compartments rather than within the cells. The appearance of global transmural and subendocardial LGE is characteristic of CA and is a predictor of prognosis.^87^ Transmural enhancement patterns are particularly helpful in differentiating ATTR from AL.^88^ However, the LGE pattern may vary and be atypical and patchy in people with early disease and exhibits poor sensitivity for diagnosis.^89–91^ Non-ischaemic patchy or mid-wall LGE is seen in about one-third of individuals with severe AS, which is linked to impaired cardiac function and unfavourable clinical outcomes.^92,93^ Therefore, the diagnosis may be challenging as patients with dual pathology may present with varied CMR-LGE combinations.^2^ For patients with suspected CA, irrespective of AS, the LGE approach has limitations as many of them have severe renal impairment, making it difficult to provide gadolinium-based contrast. Additionally, amyloid accumulation in the interstitium may blur the distinction between the blood and the myocardium, resulting in a normal appearance of the myocardium on LGE imaging despite the heart as a whole being diseased.^85^ Furthermore, the deposition of amyloid fibrils occurs in overlapping stages ranging from minimal to transmural, resulting in a lack of late enhancement in the early stages of the disease. Therefore, limiting CMR protocols to LGE imaging may overlook the early stages of CA.

The measurement of ECV represents the volume that is not occupied by cells, including intravascular and interstitial space. Extracellular volume makes up approximately 25% of myocardial volume, and it can be calculated for a specific region or set of voxels by co-registering native and post-contrast T1 images, quantifying and adjusting for haematocrit. Thereafter, an ECV map can also be produced. A high ECV value indicates excessive collagen deposition or fibrosis, which is commonly seen in amyloidosis and myocardial infarction. Both native T1 and ECV values are higher in ATTR than in HCM and have strong diagnostic accuracies for ATTR-CA, but only ECV was independently predictive of prognosis, suggesting that ECV is a more reliable diagnostic for cardiac ATTR.^94,95^ In yet another investigation, CA was observed to have considerably higher native T1 and ECV than HCM and controls.^96^ In addition to these findings, it has been demonstrated that patients with AL-CA who have greater T1 and ECV readings had shorter event-free survival.^94^ Cavalcante *et al*.^51^ also assessed ECV values in patients with dual pathology and showed higher ECV values in patients with AS-CA rather than in lone AS ones (mean ECV 41.2% ± 16.7 vs. 27.9% ± 4.1; *P* < 0.001). Native T1 and ECV alterations can both help in earlier disease identification, even before LGE is detected.^2,85,91,94^ Nitsche *et al*.^36^ confirmed the low sensitivity of distinctive LGE patterns in patients with combined clinical entities and showed that ECV increased the CMR power of discrimination (AUC: 0.756) to differentiate AS from AS-CA. Extracellular volume, however, is considered to be superior to T1 because it is specific to the extracellular environment, less susceptible to oedema, and is thought to be a direct indicator of the amyloid burden. Native T1 lacks specificity for the myocardial interstitium, the gadolinium partition coefficient in LGE is susceptible to haematocrit changes, and post-contrast T1 remains susceptible to variation in weight-based contrast dosing, the timing of post-contrast image acquisition, renal function, and haematocrit. Extracellular volume, in contrast, is immune to these confounders.^97^ In addition, since the ECV computation is based on the ratio of change in myocardial T1 relative to blood pool T1 pre- and post-contrast, it reduces the variability of test results. In contrast to native T1, ECV appears to be independent of field strength, although, like native T1, it fluctuates between systole and diastole.^85^

Whilst CMR parameters have proved to be useful for diagnosis, standard mapping approaches for T1 and T2 mapping require separate acquisitions and are influenced by various factors such as scanner technologies, pulse sequences, and physiological and environmental conditions. Moreover, the use of a gadolinium contrast agent is necessary for LGE and post-contrast T1 mapping, which is not routinely performed in many institutions. Hence, a non-contrast CMR approach that is reproducible and can accurately diagnose CA in the setting of AS whilst providing a quantitative evaluation of myocardial amyloid load would be highly advantageous.

### Radionuclide bone scintigraphy

Radionuclide studies play an exceptional role in the non-invasive diagnosis of CA. Three main groups of radioisotope-labelled agents are used to identify amyloid deposition: Iodine-123-(^123^I)-labelled serum amyloid P component (SAP), technetium-99 m (^99m^Tc)-labelled aprotinin, and ^99m^Tc-labelled bone agents. Scintigraphy or single-photon emission computed tomography (SPECT) using bone-avid tracers has demonstrated high sensitivity and specificity for ATTR-CA.^98,99^ A significant additional benefit of radionuclide testing in CA is the contemporaneous whole-body imaging that permits multi-organ evaluation. Positron emission tomography (PET) or SPECT tracers have a high affinity for amyloid fibrils and can be used to monitor the extent of systemic involvement in both AL and ATTR, although they have little role in diagnosing CA due to poor image quality.^98^ Dysautonomia and angina are frequent observations in patients with CA. Myocardial denervation imaging using ^123^I-meta-iodobenzylguanidine (mIBG) and myocardial perfusion imaging have gained interest in recent years.

### Bone-avid radiotracers for cardiac scintigraphy

Over the past decade, ^99m^Tc-labelled radiotracers have emerged as reliable diagnostic tools for ATTR-CA. Amongst them, three radiotracers are most commonly used—pyrophosphates (PYPs), bisphosphonates (DPDs), and hydroxymethylene diphosphonate (HMDP). They were conventionally used in bone scans, hence being widely known as ‘bone-avid radiotracers’, and the technique is called ‘bone scintigraphy’. Amongst other target binding sites, calcium and its microdeposits have been found to be the preferred sites that help during the diagnosis of CA,^100^ as the protein that holds amyloid fibrils together, known as amyloid P, is calcium dependent. Although all types of amyloid have the P component, which may lead to calcium diphosphonate binding in amyloid fibrils,^101^ the approach has shown a difference in its diagnostic sensitivity towards different types of CA. As ATTR-CA has a higher density of calcium deposits than AL-CA,^102^ bone-avid radiotracers show selectivity towards ATTR-CA than AL-CA. Another radiotracer—^99m^Tc-labelled methylene diphosphonate (MDP)—though commonly used in bone scintigraphy, does not show any significant degree of myocardial uptake. Hence, MDP is not the radiotracer of preference for the diagnosis of CA.^103,104^

Grading of CA using bone-avid radiotracers is done by visual comparison and semi-quantitative analysis^105^ either of which may be adopted once cardiac uptake is confirmed. Depending on the radiotracer used, planar and SPECT images are obtained at 1 and 3 h after a Tc-99m-PYP injection. In the former approach, a visual assessment of cardiac uptake on the planar and SPECT images is done (either both tests are applied parallelly, or SPECT is used as a triage test if the planar comes positive).^99^ The visual scoring system is obtained based on comparisons between cardiac uptake and bone uptake (usually in the sternum or on the rib) on images obtained from imaging techniques. A qualitative scoring system has been defined based on the relative uptake—Grade 0 is no cardiac uptake and normal bone uptake; Grade 1 reveals mild cardiac uptake that is less in intensity than bone uptake; Grade 2 shows a moderate cardiac uptake with reduced bone uptake; and Grade 3 is when the images show dense cardiac uptake with mild/absent bone uptake (*Table 1*). A grade of 2 or 3 on the qualitative scale of visual comparison is strongly suggestive of ATTR-CA. The second approach includes semi-quantitative analysis by comparing the retention of radiotracers in the heart vs. in the entire body in 5-min (early) and 3-h (late) imaging.^106^ Another and more common method is to compare the retention of radiotracer in the heart vs. in the contralateral lung (H/CL) in 1-h imaging.^107^ In the anterior planar images, an ellipse or circular region of interest (ROI) is drawn over the heart with caution to avoid sternal overlap and with size adjustments to maximize the inclusion of the heart without including the lungs in the scan. To account for the uptake of radiotracers by ribs and the background, this ROI is mirrored over the contralateral chest. The H/CL ratio is the ratio of the heart ROI mean counts and the contralateral chest ROI mean counts. For ATTR-CA, the value of H/CL is considered positive if it is greater than 1.5, whereas a value of less than 1.5 is regarded as negative.

**Table 1:** Qualitative grading of scintigraphy on the basis of visual assessment

Qualitative grading on the basis of visual assessment	
Grade	Cardiac uptake of tracer in contrast to bone uptake
Grade 0	No cardiac uptake; normal bone uptake
Grade 1	Mild myocardial uptake that is less intense than bone uptake
Grade 2	Moderate myocardial uptake; attenuated bone uptake
Grade 3	Myocardial uptake greater than bone uptake; mild/absent bone uptake

In a multi-centric study of 1217 CA patients, bone scintigraphy was found to show >99% sensitivity for ATTR-CA. Moreover, in cases with a negative UPEP or SPEP result for AL-CA, the specificity of bone scintigraphy in Grade 2 and 3 patients increased to 100%.^108^ Another study showed that all patients of severe AS exhibiting ‘red flags’ demonstrated as positive for wild-type ATTR-CA on bone scintigraphy, which was also confirmed by endomyocardial biopsy.^58^  ^99m^Tc-DPD uptake by heart has also been found in asymptomatic ATTR-CA cases,^109^ hence serving as a suitable diagnostic test even in the early stages of the disease. However, Scully *et al*.^110^ showed the presence of skewness in the amyloid load, with Grade 2 being most prevalent amongst patients with concomitant AS. This deviation from the regular normal distribution of Grade 1 > 2 > 3 suggests an interaction between the two pathologies. In addition to diagnostics and disease staging, radiotracer uptake has also proved its usefulness in prognostics and prediction of adverse cardiac events.^106^ With enough evidence of the utility of these techniques in the investigation of CA, standard protocols must be devised for incorporating these techniques into clinical practice and making them feasible for the general public.

### Amyloid-binding radiotracers

Single-photon emission computed tomography and PET are two imaging methods currently in use for the diagnosis of CA. Positron emission tomography radiotracers similar in structure to thioflavin (^18^F-florbetapir, ^18^F-florbetaben, ^11^C-PiB, and ^18^F-flutemetamol) were initially approved for imaging Alzheimer’s disease but are also able to differentiate between AL- and ATTR-CA on the basis of imaging. In 2014, Dorbala *et al*.^111^ demonstrated the utility of 18F-florbetapir for imaging and comparing AL- with ATTR-CA. Nine confirmed CA patients showed a significant increase in ^18^F-florbetapir compared with five controls. Park *et al*.^112^ further demonstrated its higher uptake in AL compared with ATTR. In another study, 14 individuals (5 AL-CA, 5 ATTR-CA, and 4 hypertensive controls) were recruited to undergo PET imaging and TTE.^113^ Increased ^18^F-florbetaben uptake was observed in all CA patients and none in hypertensive controls. Furthermore, Kircher *et al*.^114^ showed consistency in ^18^F-florbetaben retention in 14 out of 22 patients with a characteristic retention pattern (AL > AA > ATTR).

Antoni *et al*.^115^ demonstrated levels of ^11^C-PiB in 10 AL and ATTR-CA patients and compared their levels in five healthy volunteers. Retention of this tracer was found in all CA patients, whereas no uptake was seen in healthy individuals. Similar results were seen in another study where in 3/15 biopsy-proven patients, uptake of ^11^C-PiB was found, whereas none in non-CA patients.^116^ Dietemann *et al*.^117^ also determined ^18^F-flutemetamol tracer levels noted in 8/9 CA patients, whereas none in all three controls in this study. Despite the above studies showing the potential of amyloid-binding radiotracers in diagnosing CA, there are no standardized guidelines for using this technique in staging or prognosis of the disease.

## Management of amyloidosis and underlying aortic stenosis

**Figure 4:**
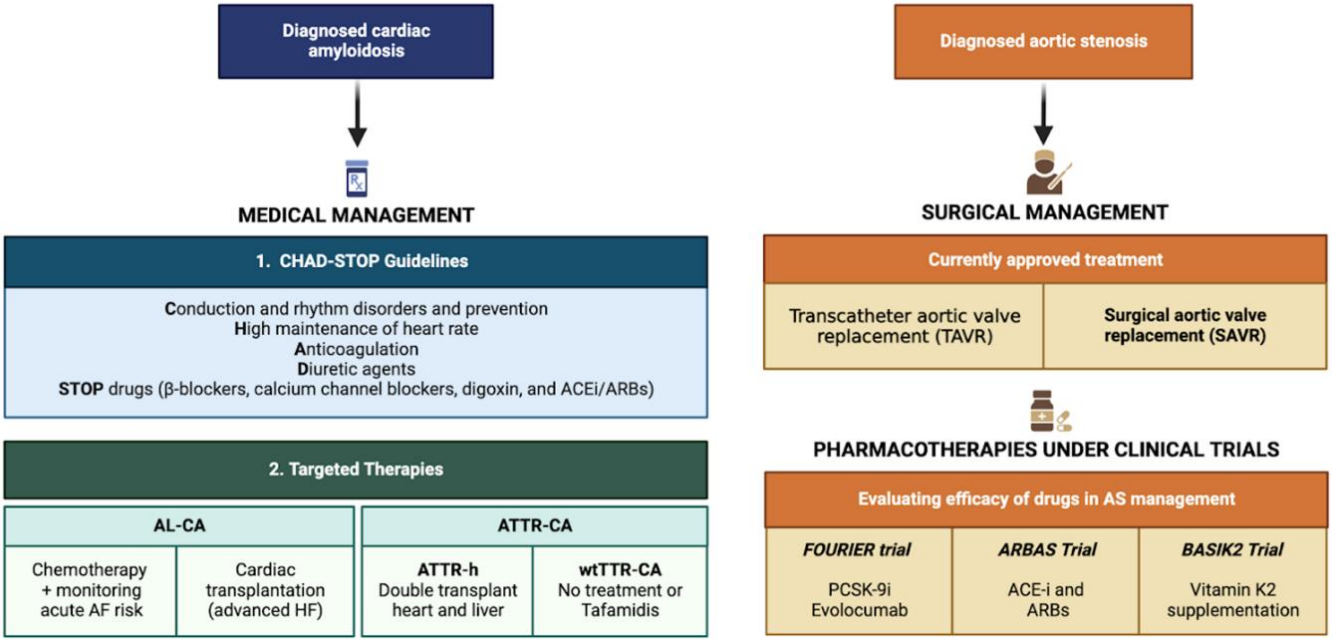
Current medical/surgical management of cardiac amyloidosis and aortic stenosis with relevant clinical trials ongoing for pharmacotherapies. ACEi, angiotensin convertase enzyme inhibitors; AL-CA, immunoglobulin light chain amyloidosis; ARBs, angiotensin receptor blockers; ATTR-CA, transthyretin cardiac amyloidosis; ATTR-h, hereditary transthyretin amyloidosis; PCSK-9i, proprotein convertase subtilisin/kexin type 9 serine protease inhibitors; wtTTR-CA, wild-type transthyretin cardiac amyloidosis.

With a significant incidence of concomitant AS and CA being found in patients, there is ambiguity as to which disease should be considered the primary prognosticator. Some studies have found comparable mortality amongst patients of either of the two pathologies and those having dual pathology—patients of ATTR-CA with and without AS were found to have similar mortality rates^118^; conversely, mortality amongst patients of lone AS was similar to patients having coexisting CA.^119^ This indicated either of the two pathologies may be the primary determinant of the outcome. However, more evidence suggests that the coexistence of both diseases has a poorer prognosis than AS alone^36,43,51^ or ATTR-CA alone^120^ in terms of mortality. Hence, there might be an interplay between the two diseases causing cardiac remodelling or collegial worsening of cardiac activity, thereby affecting mortality.^121^ Either or both pathologies can be managed by medical or surgical methods (as summarized in *Figure 4*).

Studies suggest that in cases of coexistent ATTR-CA and AS, the overall effect on the heart resembles amyloidotic pathology.^122^ After the treatment of stenosis, the dual pathology has been found to solely resemble amyloidosis.^123^ As such, after treatment of the stenotic valve, TTR-specific therapies are prescribed. With advanced HF being one of the primary complications of amyloidosis, its timely diagnosis and staging help in better downstream management of the condition. Two mainstay treatment options are heart transplant and mechanical circulatory support (MCS), either of which may be chosen on the basis of prognostic markers and available resources. Whilst amyloidotic cardiomyopathy has been given high priority in allocation of donor organs, MCS can be used as an alternative therapy in case of an unavailable donor heart or when a heart transplant is contraindicated. Palliative management may be given to patients, especially in severe and terminal stages of the disease, so as to improve the quality of life.^124^

### Medical management

The medical management of amyloidosis encompasses two aspects—targeted treatment to address amyloid deposition and general treatment to prevent disease complications. The general management of CA primarily focuses on halting or reducing the progression of HF and minimizing its effects in symptomatic patients.^7,16^ In cases of decompensated HF, diuretic agents are recommended to reduce volume overload. Amiodarone is the primary treatment for arrhythmia, whilst anticoagulants are used mainly for supraventricular arrhythmia and thrombogenic complications. However, in cases of restrictive cardiomyopathy resulting from CA, the ventricular filling is compromised, necessitating an increase in heart rate to maintain adequate cardiac output.^16^ Consequently, drugs that reduce heart rate, such as calcium channel blockers and β-blockers, are contraindicated in these patients.

Targeted treatment for AL amyloidosis involves anti-plasma cell chemotherapy, which suppresses plasma cells, leading to a decrease in the formation of light chains and reducing their toxic effects. However, a recent perspective on treating the disorder involves a multi-disciplinary approach to decrease organ damage. Regular assessment of haematological and organ response, primarily to heart and kidney function, precedes a risk stratification-driven process of modifying chemotherapy doses and subsequent transplant options. Supportive therapy has also been found useful in decreasing organ dysfunction, particularly in cases with advanced heart disease.^108^

For ATTR-CA, targeted therapy utilizes three mechanisms: decreasing the production of misfolded TTR from the liver, preventing the dissociation of TTR tetramer into subsequent forms, and removing amyloid deposition from cardiac tissue.^7^ The drug of choice is tafamidis, which acts as a TTR stabilizer. It binds to the thyroxin-binding site of TTR, preventing the dissociation of TTR to form amyloid fibrils. In the Transthyretin Amyloidosis Cardiomyopathy Clinical Trial (ATTR-ACT), patients being administered tafamidis showed a decrease in hospitalization due to cardiovascular causes and all-cause mortality. The decline in quality of life and functional capacity were significantly less in patients being administered tafamidis than placebo.^125^

Since tafamidis inhibits disease progression but may not always cause it to regress, its administration in the early stages of the disease is associated with greater efficacy. With the progression of the disease, administration of TTR stabilizers in the later stages of the disease leads to higher mortality either due to progressive HF or other causes.^126^ As such, early diagnosis of the disease, whether presenting as a sole pathology or with any other underlying illness, expedites the process of treating it, thereby improving outcomes. A secondary model based on the ATTR-ACT showed that tafamidis is projected to add 1.29 quality-adjusted life years (QALY) in comparison with conventional treatment. However, cost-effectiveness analysis in the same model revealed that a 92.6% price decrement is required for tafamidis to be effective at $100 000/QALY.^127^ With only mild to moderate side effects and minimal safety monitoring requirements, tafamidis has shown great potential in becoming the mainstay treatment for ATTR-CM.

Although medical treatment in the case of concomitant AS and ATTR-CA is primarily directed towards amyloid pathology, it becomes imperative to treat the effects of the valvular disease in cases of advanced AS leading to acute decompensation and HF. The main aim of drug therapy in this scenario is to stabilize the haemodynamic profile of the patient.^128^ Whilst loop diuretics are the mainstay treatment in HF for haemodynamic stabilization, their benefit in the setting of AS is limited by low intravascular volume and reflex vasoconstriction response, causing hypoperfusion. Tolvaptan, an oral selective V2 receptor antagonist, has been proved safe and efficacious in causing net volume depletion in cases of acute HF and severe AS.^129,130^ Inotropes and vasopressors, traditionally used to treat acute HF by increasing tissue perfusion, are being progressively limited due to side effects such as arrhythmias and myocardial injury.^131^ A calcium stabilizer by the name of levosimendan, having inotropic and vasodilator properties, has been shown to improve the symptoms and haemodynamic profile of patients in acute HF with severe AS.^132^ In spite of substantial potential medical therapies in this setting, valve replacement stays the singular most definitive management of the condition.^133^

### Surgical management

Patients with cardiomyopathy secondary to AL-CA or ATTR-CA who have responded to light chain therapy may be given the option of a heart transplant.^134^ Whilst patients with ATTRwt or ATTRv-CA Val122Ile mutation require only heart transplantation, other forms, such as Thr60Ala, may also need liver transplants besides heart transplants.^134^ Heart transplantation for CA is associated with major risks, like recurrence in cardiac allografts leading to a reduction in 1- and 5-year post-transplant survival rates.^135^ However, the studies conducted by Lacy *et al*.^136^ and Dey *et al*.^137^ showed that the survival rate had been improved by the implantation of post-heart transplant autologous haematopoietic stem cell transplant and light-chain reductive chemotherapy.

The surgical treatment options for patients with severe AS are TAVR or surgical AVR (SAVR), depending on patient characteristics.^138^ A few studies have assessed the efficacy of TAVR and SAVR in patients with AS and CA. A study by Galat *et al*.^59^ showed a better outcome for patients with AS-CA with TAVR than SAVR, though the studies contained only a few participants (*n* = 16). Similar results were shown by a national inpatient sample conducted by Khan *et al*. They revealed higher mortality and hospitalization cost amongst those patients who underwent SAVR compared with those who underwent TAVR after propensity score matching in diagnosed CA with AS patients.^139^ Transcatheter aortic valve replacement has also been shown to have higher efficacy than medical therapy in CA with severe AS patients. Cannata *et al*.^140^ conducted a meta-analysis, which showed a lower risk of overall cause mortality in patients who underwent TAVR compared with those who were administered medical therapy alone. Similarly, another study showed a lower rate of hospital re-admissions and all-cause mortality after undergoing TAVR in CA with AS patients compared with those treated with medical therapy only.^141^ In yet another study of 191 patients with degenerative AS scheduled for TAVR, 16 cases were found to have CA.^36^ Despite this finding, the presence of CA did not show any significant negative impact on outcomes during a median follow-up of 15.3-month post-TAVR.^36^ Another study investigating TAVR outcomes in patients with existing CA also revealed no significant difference in mortality and 30-day hospital readmission rates compared with the control group.^142^ However, it did indicate a higher rate of ischaemic stroke in amyloidosis patients, suggesting a potential association with increased thromboembolic risk following TAVR.^142^ Whilst some researchers suggest that TAVR patients might benefit from disease-modifying therapy for CA,^143^ no dedicated randomized trial has been conducted yet, making it a highly desirable area for future investigation.

Once CA is diagnosed in AS patients, the different treatment options, i.e. medical management or valvular replacement (TAVR or SAVR), should be discussed by multi-disciplinary heart teams, including cardiomyopathy specialists and geriatricians. Various factors are associated with poor prognosis with the futility of AVR, e.g. low LVEF < 50%, restrictive pattern (Grade III diastolic dysfunction), severely reduced GLS (≥−10%), low-gradient AS, and moderate to severe low flow state (SVi < 30–35 mL/m^2^).^16^ These factors, along with other concomitant conditions, the functional status of the patient, life expectancy, and frailty, should be taken into account to select the best therapeutic options for the patients.

## Conclusion

Cardiac amyloidosis is highly associated with AS in the geriatric population. These two conditions have common characteristics and worsen the prognosis when they occur together. Amyloid infiltration in the aortic valve can develop and/or worsen the stenosis. Multi-parametric assessment is the key to diagnosing CA in patients with AS. In case of severe stenosis, valve replacement remains the best approach for beneficial outcomes. Further studies are needed to define the prognosis and the correct therapeutic pathway for patients with CA and AS.

## Data Availability

The data underlying this article are available in the article or will be available upon request from lead author Vikash Jaiswal.

## Lead author biography

Vikash Jaiswal is a Doctor of Medicine and outcome-based cardiovascular researcher at Larkin Community Hospital, South Miami, FL, USA. He is interested in exploring the field of Cardio-Oncology, Disparity amongst Cardiovascular Disease outcomes, and Women's Health and Breast Cancer prevention. He won the Prestigious Paul Dudley White International Scholar Award in 2021 and 2022 from the American Heart Association for the best Cardiovascular Research from the Philippines and India region. He supports women in the field of cardiology and motivates them to participate in his cardiology research. His elder brother Dr Akash is his role model and motivates him to perform his best in the medical field.
